# Effect of statins on cardiovascular events in patients with mild to moderate chronic kidney disease: a systematic review and meta-analysis of randomized clinical trials

**DOI:** 10.1186/1471-2261-14-19

**Published:** 2014-02-17

**Authors:** Xiao Zhang, Chun Xiang, Yu-Hao Zhou, An Jiang, Ying-Yi Qin, Jia He

**Affiliations:** 1 Department of Health Statistics, Second Military Medical University, 200433 Shanghai, China; 2 Department of Rehabilitation Institute, Shanghai Seventh People’s Hospital, Shanghai, China; 3 Office of Educational Administration, Second Military Medical University, Shanghai, China

**Keywords:** Statin, Cardiovascular events, Chronic kidney disease, Meta-analysis

## Abstract

**Background:**

Statins are commonly used to lower total cholesterol levels in the general population to prevent cardiovascular events. However, the effects of statins in patients with chronic kidney disease remain unclear. We therefore performed a meta-analysis to assess the effects of statin therapy on cardiovascular outcomes in patients with mild to moderate chronic kidney disease.

**Methods:**

We systematically searched PubMed, EmBase, the Cochrane Central Register of Controlled Trials, proceedings of major meetings, and reference lists of articles for relevant literature. Only randomized clinical trials were included. Outcomes analysed included cardiovascular disease, total mortality, myocardial infarction, stroke, cardiovascular death, and possible drug-related adverse events. Subgroup analyses were also performed based on the population characteristics and clinical indexes.

**Results:**

Twelve trials met our inclusion criteria. Overall, statin therapy resulted in a 24% reduction in the risk of cardiovascular disease (RR = 0.76,95% confidence interval [CI], 0.72– 0.80), a 21% reduction in the risk of total mortality (RR = 0.79,95% CI, 0.72–0.86), a 34% reduction in the risk of myocardial infarction (RR = 0.66,95% CI, 0.52–0.83), a 30% reduction in the risk of stroke (RR = 0.70,95% CI, 0.57–0.85), and a 17% reduction in the risk of cardiovascular mortality (RR = 0.83,95% CI, 0.73– 0.93). No statistically significant drug-related adverse events were noted. Subgroup analysis indicated that some important factors such as baseline creatinine level ≥1.5 mg/dL, baseline glomerular filtration rate (GFR), and cardiovascular disease history could affect cardiovascular outcomes.

**Conclusion:**

Statin therapy had a clear effect on cardiovascular disease, total mortality, stroke, and myocardial infarction in patients with mild to moderate renal disease. Subgroup analysis indicated that baseline GFR, baseline creatinine level, and a history of cardiovascular disease might play an important role in the cardiovascular outcomes.

## Background

Chronic kidney disease (CKD) is the progressive loss of renal function that occurs over months or even years, and is increasingly recognized as a global public health problem [1-3]. Studies have indicated that patients with CKD have cardiovascular mortality rates at least 10 times higher than that of the general population [4]. These higher rates increase disease burden and treatment cost. However, little is known about the prevention of cardiovascular disease in patients with CKD. An increasing number of experts suggest statin therapy for the prevention and treatment of cardiovascular disease in patients with CKD [5-7].

Statins can reduce blood cholesterol levels and are confirmed as the first-choice agent in the prevention and treatment of cardiovascular disease in patients with or without a history of cardiovascular disease [8]. Some clinicians were concerned about the progression of cardiovascular disease in patients with CKD and extended statin therapy to these patients. They considered CKD to be an important and independent cardiovascular risk factor, and found that patients with mild to moderate renal disease are more likely to develop cardiovascular disease than progress to end-stage kidney disease [9-12]. Laboratory studies proved that the pathophysiology of cardiovascular disease differed from the severity of CKD, and end-stage renal disease occurs too late to improve cardiovascular outcomes [13].

Many clinical trials have investigated the effects and safety of statins in patients with mild to moderate CKD; most of them provided positive results. However, some studies illustrated that statins are useful in treating mild CKD disease but ineffective for moderate CKD when patients were categorized by disease stage [14]. In addition, a meta-analysis reported the beneficial effects of statin therapy in patients with CKD [15], but did not focus on mild to moderate CKD to explore the possible influence of population characteristics, clinical features, and quality of trials. Moreover, more data were added to our studies to provide stronger evidence in consideration of adverse events. Hence, we performed a systematic review and meta-analysis to evaluate the effects of statin therapy on cardiovascular outcomes in patients with predialysis CKD, with the aim to assess the efficacy and safety of statins in patients with mild to moderate CKD.

## Methods

### Data sources, search strategy, and selection criteria

This study was approved by the Ethics Committee of the Second Military Medical University. Randomized, double-blind, controlled trials and trials of statins in the English-language literature were eligible for inclusion in our study. Relevant trials were found with the following procedure:

(1) Electronic searches: We systematically searched PubMed, EmBase, MEDLINE, and the Cochrane Central Register of Controlled Trials for articles published since December 20, 2011. Search terms used were kidney disease, renal disease, nephropathy, nephrosis, 3-hydroxy-3-methylglutaryl-coenzyme A, statin, statins, lovastatin, simvastatin, pravastatin, fluvastatin, atorvastatin, rosuvastatin, pitavastatin, cerivastatin, ulinastatin, and pentostatin. (2) Additional resources: We contacted authors by email or telephone to obtain all potentially relevant study details (published or unpublished data) regarding their trials and manually searched for proceedings of international conferences in the Cochrane Cardiovascular Disease Group Specialized Register. In addition, we complemented the search by using the PubMed “related articles” function and the science citation by scrutinizing reference lists of included studies and the cross references of relevant papers. Finally, we also searched for ongoing randomized controlled trials (RCTs) that were registered as completed but not published trials, in the Meta Register of Controlled Trials.

The inclusion criteria for the RCTs were as follows: (1) high-quality RCTs comparing any statin with a placebo, no treatment, or another statin, or RCTs comparing low-dose statins with high-dose statins; (2) RCTs including patients with mild to moderate CKD (stages 1–4) [16,17] including end-stage renal disease (estimated glomerular filtration rate [GFR] <15 mL · min^-1^ · 1.73 m^-2^), but excluding those with patients on dialysis or those who had a kidney transplant; (3) RCTs with a minimum 8-week follow-up period; and (4) those with a sample size >50. The literature search and selection were undertaken independently by two investigators (Xiao-Zhang and Chun-Xiang), and any disagreement between them was mediated by a third investigator (Yu-Hao Zhou) until consensus was reached.

### Data extraction and quality assessment

Data extraction and collection were performed independently by two reviewers (Xiao Zhang and Chun Xiang) using a standardized protocol. Any conflict between the reviewers was resolved by a group discussion, after which the primary authors (Yu-Hao Zhou and Jia He) made the final decision. The data extraction included baseline patient characteristics (age, sex, total cholesterol level, GFR, creatinine level, urinary albumin level, intervention, drug dosage, follow-up duration, and adverse events) and outcomes (cardiovascular events, stroke, myocardial infarction, total mortality, cardiovascular mortality, and possible drug-related adverse events). The quality of the included trials was assessed with the Jadad score [18] on the basis of randomization, concealment of treatment allocation, blinding, completion of follow-up, and use of intention-to-treat analysis.

### Statistical analysis

We allocated the results of each RCT as dichotomous frequency data. We compared the pooled relative risks (RRs) and 95% confidence intervals [CIs] of cardiovascular disease, total mortality, stroke, myocardial infarction, cardiovascular death, and adverse events between the statin therapy and control groups. Subgroup analyses were performed according to mean age (≥60 years or <60 years), cardiovascular history (with or without cardiovascular history), diabetes (present or absent), baseline GFR level (60–90 mL · min^-1^ · 1.73 m^-2^ or 30–60 mL · min^-1^ · 1.73 m^-2^), baseline creatinine level (≥1.5 mg/dL or <1.5 mg/dL), baseline total cholesterol level (≥5.0 mmol/L or < 5.0 mmol/L), baseline low-density lipoprotein (LDL) cholesterol level (≥3.0 mmol/L or <3.0 mmol/L), LDL cholesterol lowering rate (≥30% or <30%), and follow-up duration (≥3 years or <3 years) to investigate the change in the risk estimates with population characteristics and clinical indexes. Both the fixed-effects and random-effects models were used to evaluate the pooled RRs and 95% CIs for the effects of statin therapy. Although both models yielded similar findings, results from the random-effects model indicated that the true underlying effect varied among the included trials and seemed more conservative [19,20]. Heterogeneity of treatment effects between trials was evaluated by the Q test and I-squared (I^2^) statistic [21]. These indexes assess the percentage of variability across studies that are attributable to heterogeneity rather than chance. Statistical heterogeneity was considered significant when p < 0.10 for the chi-square test or I^2^ > 50%. Several methods were used to check for potential publication bias on the outcomes of cardiovascular disease, total mortality, stroke, myocardial infarction, and cardiovascular mortality. Visual inspection of the funnel plot was performed. The Peters test was used to statistically analyse publication bias [22]. We also conducted a sensitivity analysis by removing each individual study from the meta-analysis. All reported p values were two-sided and p values <0.05 were regarded as statistically significant. Statistical analyses were performed using the STATA 11.0 (State Corporation, Lake Way, Texas, USA).

## Results

### Trial characteristic

**Figure 1:**
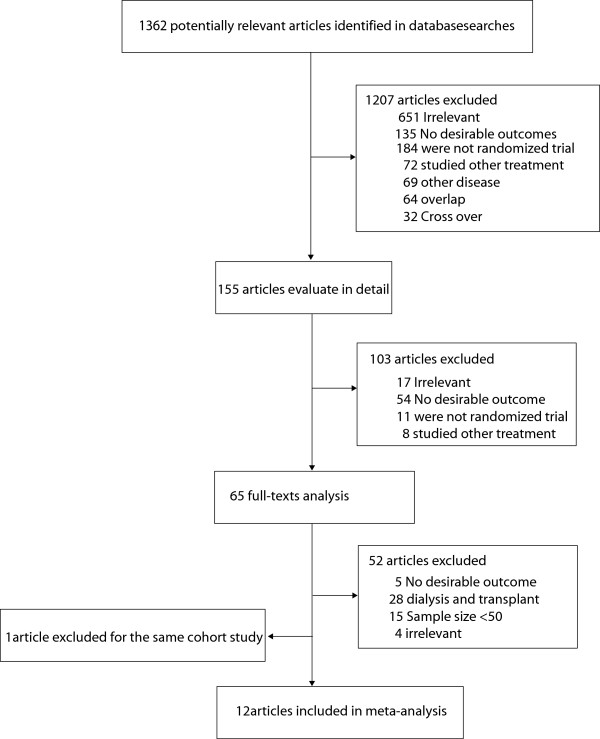
Flow diagram of the literature search and selection process.

Twelve RCTs were included in our meta-analysis (Figure 1) [14,23-34]. One study was reported in two separate published articles [24,31], and only the recent one was included in our analysis [31]. Four trials [23-26] evaluated the effect of pravastatin, three studies [14,27,28] evaluated the effect of simvastatin, two trials [29,30] evaluated the effect of atorvastatin, one study [31] evaluated the effect of fluvastatin, one study [32] evaluated the effect of lovastatin, and the remaining study [33] evaluated the effect of rosuvastatin. The quality of the trials was assessed using the Jadad score. Overall, two trials had a Jadad score of 5, one trial had a score of 4, three trials had a score of 3, and the remaining six trials had a score of 2. Details on each study are listed in Table 1.

**Table 1:** Characteristic of studies included in the meta-analysis

**Study**	**Intervention**	**Control**	**No. of patient**	**Gender (female)**	**Mean age, y**	**Co-morbidity of CVDs**	**Co-morbidity of diabetes**	**Total cholesterol (mmol/L)**	**GFR (ml/min/1.73 m**^**2**^**)**	**Creatinine (mg/dL)**	**LDL lowing**	**Follow-up (month)**	**Jadad score**
CARE(2003)^34^	Pravastatin	Placebo	1711	369(21.6%)	64.3	AMI	NR	5.40	NR	1.26	NR	58.9	3
HPS(2003)^27†^	Simvastatin	Vitamins/Placebo	1329	NR	NR	NR	NR	NR	NR	NR	NR	57.0	2
PPP(2004)^23^	Pravastatin	Placebo	16824	1840(10.9%)	59.5	NR	NR	6.05	68.5	1.18	51.1%	60.0	3
PREVENDIT (2004)^24^	Pravastatin	Fosinopril/Placebo	864	303(35.1%)	51.3	NR	3%	5.80	NR	1.02	24.4%	46.0	4
LIPIS(2005)^31^	Pravastatin	Placebo	310	102(33%)	69.0	PCI	NR	5.17	NR	1.33	NR	46.0	2
4S Study (2007)^28^	Simvastatin	Placebo	2314	618(26.7%)	60.5	CHD	NR	6.75	65.2	1.5	38.0%	65.5	2
ATIC(2007)^25^	Pravastatin	Placebo	93	40(43.0%)	53.0	No history of AOD	Excluded diabetes	5.60	33.5	2.32	26.1%	18.0	5
TNT(2008)^30^	Atorvastatin	Atorvastatin	3107	1005(32.3%)	65.5	CHD	NR	4.55	52.9	NR	18.0%	60.0	2
AFCAPS (2009)	Lovastatin	Placebo	304	65(21.4%)	62.0	No history of CVD	Excluded diabetes	5.74	53.0	1.4	27.0%	64.0	3
ALLIANCE (2009)^29^	Atorvastatin	Usual Care	579	134(23.1%)	65.2	CHD	NR	5.89	51.2	1.5	34.5%	54.3	2
MEGA(2009)^26^	Pravastatin	Diet	7196	NR	NR	No history of CVD	NR	NR	65.0	NR	18.5%	64.0	2
JUPITER (2010)^33^	Rosuvastatin	Placebo	3267	2129(65.2%)	70.0	NR	Excluded diabetes	4.89	56.0	NR	52.0%	22.8	2
SHARP (2011)^14^	Simvastatin	Placebo	6247	NR	NR	No history of MI	NR	NR	NR	NR	68.0%	58.8	5

*Abbreviations*: *CVD* cardiovascular disease, *LDL* low density lipoprotein, *AMI* acute myocardial infarction, *PCI* percutaneous coronary intervention, *CHD* coronary heart disease, *AOD* arterial occlusive disease, *MI* myocardial infarction, *NR* not reported, *GFR* Glomerular filtration rate. ^†^: only a subgroup of the total study was included.

### Total effect

**Figure 2:**
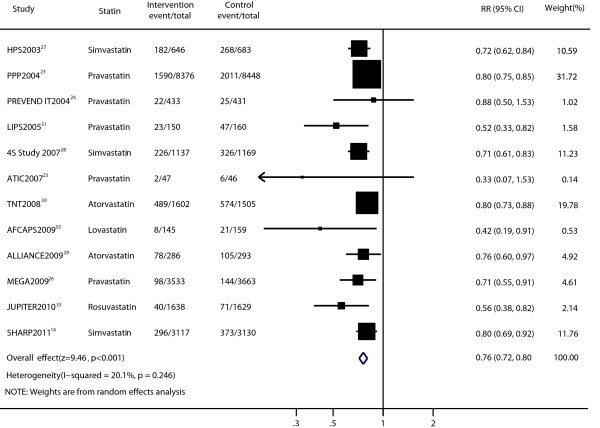
Forest plot of the effect of statin on cardiovascular disease in patients with mild to moderate chronic kidney disease.

Data for the effect of statin therapy on cardiovascular disease were available from 12 trials [14,23,25-34]. Statin therapy produced a 24% reduction in the risk of cardiovascular disease (RR, 0.76; 95% CI, 0.72–0.80; p < 0.001; Figure 2) using a random-effects model, and significant heterogeneity was not observed among individual trials (p = 0.246; I^2^ = 20.1%). Sensitivity analyses showed that the RR and 95% CI were not substantially altered after removal of any single study (data not shown). The results of the Peters test indicated potential publication bias (p = 0.005; Additional file 1: Figure S1a). However, the results of the trim and fill method showed that no trimming was performed and that the pooled result was not changed.

**Figure 3:**
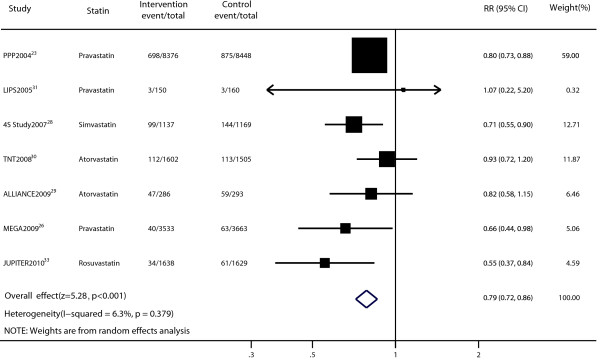
Forest plot of the effect of statin on total mortality in patients with mild to moderate chronic kidney disease.

Data for the effect of statin therapy on total mortality were available from seven trials [23,26,28-31,33]. The pooled results revealed that total mortality was significantly lower among patients undergoing stain therapy (RR, 0.79; 95% CI, 0.72–0.86; Figure 3) by using a random-effects model. No significant heterogeneity was noted (p = 0.379; I^2^ = 6.3%). Sensitivity analyses showed that the RR and 95% CI were not substantially altered after removal of any single study (data not shown). We found no evidence of publication bias in the pooled analysis of total mortality by using the funnel plot and Peters test (p = 0.984; Additional file 1: Figure S1b).

**Figure 4:**
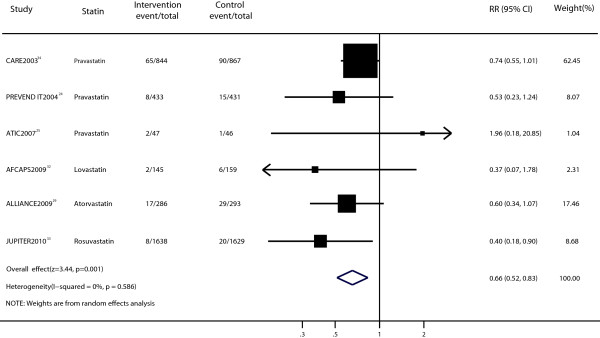
Forest plot of the effect of statin on myocardial infarction in patients with mild to moderate chronic kidney disease.

Six trials provided the data for myocardial infarction [25,29,32-35]. The pooled RR for the effect of myocardial infarction in patients receiving statin therapy compared with that of the control treatment was 0.66 (RR, 0.66; 95% CI, 0.52–0.83; p = 0.001; Figure 4). There was no evidence of significant heterogeneity among individual studies (p = 0.586; I^2^ = 0%). Sensitivity analyses showed that the RR and 95% CI were not substantially altered after removal of any single study (data not shown). No publication bias was found using the Peters test (p = 0.618; Additional file 1: Figure S1c).

**Figure 5:**
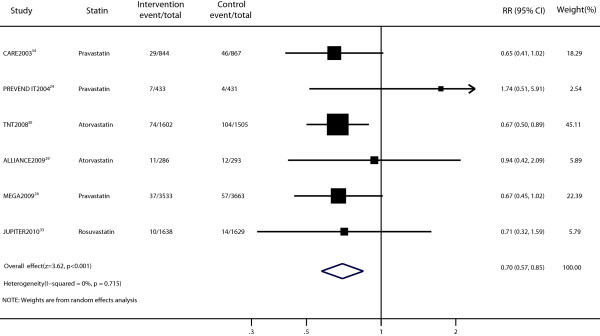
Forest plot of the effect of statin on stroke in patients with mild to moderate chronic kidney disease.

The effect of statin therapy on stroke was presented in six trials [26,29,30,33-35]. The RR was statistically significant in the two groups (RR, 0.70; 95% CI, 0.57–0.85; p < 0.001; Figure 5) with no significant heterogeneity (p = 0.715, I^2^ = 0%). Sensitivity analyses showed that the RR and 95% CI were not substantially altered after removal of any single study (data not shown). No evidence of significant publication bias was observed (Peters test, p = 0.167; Additional file 1: Figure S1d).

**Figure 6:**
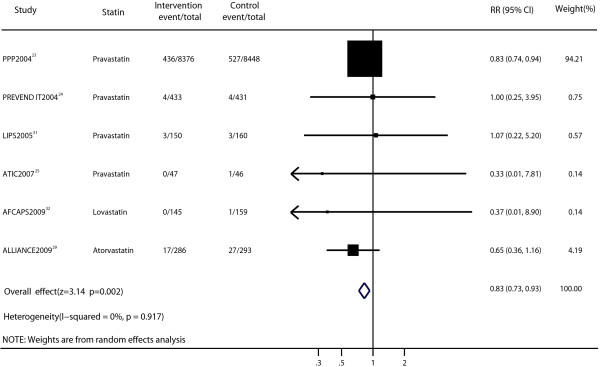
Forest plot of the effect of statin on cardiovascular mortality in patients with mild to moderate chronic kidney disease.

The efficacy of statin therapy for the risk of cardiovascular mortality was presented in six trials [23,25,29,31,32,35]. The results indicated a statistically significant decrease in the risk of cardiovascular death (RR, 0.83; 95% CI, 0.73–0.93; p = 0.002; Figure 6) with no significant heterogeneity (p = 0.917, I^2^ = 0%). However, after excluding the trial of PPP [23], the outcome was not significant (RR, 0.70; 95% CI, 0.42–1.14). No evidence of significant publication bias was observed (Peters test, p = 0.539; Additional file 1: Figure S1e).

### Toxicities

**Figure 7:**
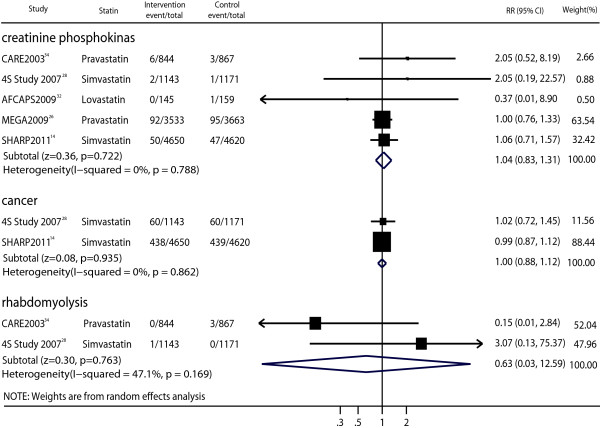
Forest plot of the effect of statin on possible drug-related adverse events in patients with mild to moderate chronic kidney disease.

Five trials reported data on patients who developed elevated serum creatine phosphokinase levels >3 or 10 times the upper limit of the normal range [14,26,28,32,34]. No significant differences were seen in the treatment and placebo groups (RR, 1.04; 95% CI, 0.83–1.31; p = 0.722; Figure 7) with no heterogeneity (p = 0.788, I^2^ = 0%).

Two studies provided data on cancer [14,28], and the pooled analysis showed no significant differences for cancer between the statin therapy and control groups (Figure 7). The adverse effect of statin therapy on rhabdomyolysis could be assessed in two trials [28,34], and the pooled analysis also showed no significant differences for cancer between patients receiving statin therapy and those receiving the placebo (Figure 7).

### Subgroup analysis

Superior efficacy of statin therapy on cardiovascular events was found in the majority of subgroup analyses, except for patients with a baseline creatinine level ≥1.5 mg/dL (RR, 0.71; 95% CI, 0.45–1.12; p = 0.139; Table 2). We noted that statin therapy had no effect on total mortality in patients with a history of cardiovascular disease (RR, 1.48; 95% CI, 0.41–5.36; p = 0.55), baseline GFR <60 mL · min^-1^ · 1.73 m^-2^ (RR, 0.68; 95% CI, 0.47, 1.00; p = 0.050), and baseline creatinine level >1.5 mg/dL (RR, 0.82; 95% CI, 0.58–1.15; p = 0.251; Table 3). Additional factors such as age, cardiovascular history, baseline creatinine level, LDL cholesterol lowering rate, and follow-up duration also showed some heterogeneity between groups in the subgroup analysis of myocardial infarction (Table 4). Most of the subgroup analyses on stroke were not effective, except for a follow-up duration >3 years (Table 5). None of the subgroup analyses on cardiovascular death was statistically significant (Table 6).

**Table 2:** Subgroup analysis for the effect of statin therapy on cardiovascular disease

**Subgroup**	**Value**	**Reference**	**Statin**	**Control**	**Relative risk (95% CI)**	**p value**	**Heterogeneity**	**p value for heterogeneity**
			**(Event/total)**	**(Event/total)**				
Mean Age	> = 60	14,27-29,31-33	853/7119	1211/7223	0.72 [0.65, 0.79]	<0.001	21.2%	0.27
	<60	23-25	1614/8856	2042/8925	0.80 [0.75, 0.84]	<0.001	0%	0.50
Cardiovascular History	Yes	28-29,31,34	498/2417	712/2489	0.72 [0.65, 0.80]	<0.001	0%	0.49
	No	14,25-26, 32	404/6842	544/6998	0.73 [0.60, 0.88]	0.001	27.6%	0.25
Diabetes	Yes	27-28,34	173/1334	260/1424	0.74 [0.63, 0.88]	<0.001	0%	0.94
	No	25,27-28,32-34	329/3123	484/3129	0.68 [0.59, 0.79]	<0.001	10%	0.35
Baseline GFR	> = 60	23,26,28	1914/13046	2481/13280	0.77 [0.71, 0.83]	<0.001	21.9%	0.28
	<60	25,29, 32-33	128/2116	203/2127	0.62 [0.47, 0.83]	0.001	29.4%	0.24
Baseline Creatinine	> = 1.5	25,29	80/333	111/339	0.71 [0.45, 1.12]	0.139	11.6%	0.29
	<1.5	23,24,28,31-32	1869/10241	2430/10367	0.73 [0.63, 0.84]	<0.001	48.0%	0.10
Baseline Total Cholesterol	> = 5.0	23,28	1816/9513	2337/9527	0.77 [0.70, 0.84]	<0.001	35.6%	0.21
	<5.0	24-25,29,31-34	344/3543	509/3585	0.69 [0.60, 0.80]	<0.001	18.1%	0.29
Baseline LDL Cholesterol	> = 3.0	23-25,27-29, 31-32	2131/11220	2809/11389	0.74 [0.70, 0.81]	P < 0.001	29.0%	0.20
	<3.0	14,30,33	825/6357	1018/6264	0.76 [0.72, 0.81]	P < 0.001	37.7%	0.20
LDL Cholesterol Lowering	> = 30%	14,23,26,28,29,33	2328/18087	3030/18332	0.77 [0.73, 0.82]	P < 0.001	10.4%	0.35
	<30%	24,25,32	32/625	52/636	0.76 [0.71, 0.82]	0.087	35.4%	0.21
Follow Up	> = 3	14,23-24, 26–29,31-32	2523/17823	3320/18136	0.76 [0.72, 0.81]	P < 0.001	14.4%	0.31
	<3	25,33	42/1685	77/1675	0.74 [0.69, 0.80]	0.001	0%	0.51

*Abbreviation*s: *CI* confidential interval, *GFR* glomerular filtration rate, *LDL* low density lipoprotein.

**Table 3:** Subgroup analysis for the effect of statin therapy on total mortality

**Subgroup**	**Value**	**Reference**	**Statin**	**Control**	**Relative risk (95% CI)**	**p value**	**Heterogeneity**	**p value for heterogeneity**
			**Event/total**	**Event/total**				
Mean Age	> = 60	23	183/3211	267/3251	0.71[0.59,0.84]	<0.001	0%	0.52
	<60	28,29,31,33	698/8376	873/8448	0.81[0.73,0.89]	<0.001	-	-
Cardiovascular History	Yes	28,29,31,34	235/2417	317/11689	1.48[0.41,5.36]	0.550	98.1%	<0.001
	No	26	40/3533	63/3663	0.66[0.44,0.98]	0.037	-	-
Baseline GFR	> = 60	23,26,28	837/13046	1082/13280	0.78[0.72,0.85]	<0.001	0%	0.42
	<60	29,33	47/286	59/293	0.68[0.47,1.00]	0.050	49.8%	0.16
Baseline Creatinine	> = 1.5	23,28,31	47/286	59/293	0.82[0.58,1.15]	0.251	-	-
	<1.5	29	800/9663	1022/9777	0.79[0.73,0.87]	<0.001	0%	0.58
Baseline Total Cholesterol	> = 5.0	29,31,33,34	797/9513	1017/9617	0.75[0.62,0.90]	0.002	0%	0.45
	<5.0	23,28	282/4520	347/4454	0.79[0.67,0.93]	<0.001	0%	0.33
Baseline LDL Cholesterol	> = 3.0	23,28-29,31,34	933/10793	1192/10937	0.79[0.73,0.86]	<0.001	0%	0.89
	<3.0	33	34/1638	61/1629	0.55[0.37,0.84]	0.005	-	-
Follow-up time	> = 3	23,26,28,29,31,34	973/14326	1255/14600	0.79[0.73,0.85]	<0.001	0%	0.86
	<3	33	34/1638	61/1629	0.55[0.37,0.84]	0.005	-	-

*Abbreviations*: *CI* confidential interval, *GFR* glomerular filtration rate, *LDL* low density lipoprotein.

**Table 4:** Subgroup analysis for the effect of statin therapy on myocardial infarction

**Subgroup**	**Value**	**Reference**	**Statin**	**Control**	**Relative risk (95% CI)**	**p value**	**Heterogeneity**	**p value for heterogeneity**
			**Event/total**	**Event/total**				
Mean Age	> = 65	29,32-34	92/2913	145/2948	0.66 [0.51, 0.85]	0.001	0%	0.44
	<65	24,25	10/480	16/477	0.63 [0.27, 1.47]	0.283	3%	0.31
Cardiovascular History	Yes	29,34	82/1130	119/1160	0.67 [0.14, 3.22]	0.012	0%	0.53
	No	25,32	4/192	7/205	0.71 [0.54, 0.93]	0.613	25.1%	0.25
Baseline Creatinine	> = 1.5	25,29	19/333	30/339	0.64 [0.37, 1.12]	0.120	0%	0.34
	<1.5	24,32,34	75/1422	111/1457	0.70 [0.53, 0.93]	0.013	0%	0.55
Baseline LDL Cholesterol	> = 3.0	24,25, 29,32,34	94/1755	141/1796	0.69 [0.53, 0.88]	0.003	0%	0.704
	<3.0	33	8/1638	20/1629	0.40 [0.18, 0.90]	0.027	-	-
LDL Cholesterol Lowering	> = 30%	29,33	25/1924	49/1922	0.52 [0.33, 0.84]	0.007	0%	0.418
	<30%	24,25,32	12/625	22/636	0.55 [0.27, 1.13]	0.104	0%	0.505
Follow-up time	> = 3	24,29,32, 34	92/1708	140/1750	0.68 [0.53, 0.87]	0.003	0%	0.70
	<3	25,33	10/1685	21/1675	0.59 [0.15, 2.27]	0.443	36%	0.21

*Abbreviations*: *CI* confidential interval, *LDL* low density lipoprotein.

**Table 5:** Subgroup analysis for the effect of statin therapy on stoke

**Subgroup**	**Value**	**Reference**	**Statin**	**Control**	**Relative risk (95% CI)**	**p value**	**Heterogeneity**	**p value for heterogeneity**
			**(Event/total)**	**(Event/total)**				
Mean Age	> = 60	29,33,34	50/2768	72/2789	0.71[0.50,1.01]	0.058	0%	0.732
	<60	24	7/433	4/431	1.74[0.51,5.91]	0.373	-	-
Cardiovascular History	Yes	29,34	40/1130	58/1160	0.71[0.48,1.05]	0.088	0%	0.429
	No	26	37/3533	57/3663	0.67[0.45,1.02]	0.059	-	-
Baseline GFR	> = 60	26	37/3533	57/3663	0.67[0.45,1.02]	0.059	-	-
	<60	29,33	21/1924	26/1922	0.82[0.46,1.45]	0.488	0%	0.63
Baseline Creatinine	> = 1.5	29	11/286	12/293	0.94[0.42,2.09]	0.878		
	<1.5	24,34	36/1277	50/1298	0.90[0.36,2.23]	0.815	54.9%	0.14
Baseline LDL Cholesterol	> = 3.0	24,29,34	47/1563	62/1591	0.82[0.51,1.30]	0.387	20.3%	0.29
	<3.0	33	10/1638	14/1429	0.71[0.32,1.60]	0.407	-	-
LDL Cholesterol Lowering	> = 30%	29,33	21/1924	26/1922	0.82[0.46,1.45]	0.488	0%	0.63
	<30%	24,26	44/3966	61/4094	0.90[0.38,2.14]	0.818	52.2%	0.15
Follow-up time	> = 3	24,26,29,34	84/5096	119/5254	0.73[0.55,0.96]	0.023	0%	0.43
	<3	33	10/1638	14/1629	0.71[0.32,1.59]	0.407	-	-

*Abbreviations*: *CI* confidential interval, *GFR* glomerular filtration rate, *LDL* low density lipoprotein.

**Table 6:** Subgroup analysis for the effect of statin therapy on cardiovascular death

**Subgroup**	**Value**	**Reference**	**Statin**	**Control**	**Relative risk (95% CI)**	**p value**	**Heterogeneity**	**p value for heterogeneity**
			**(Event/total)**	**(Event/total)**				
Mean Age	> = 65	24,25	76/1425	99/1479	0.79 [0.59, 1.06]	0.114	0%	0.81
	<65	29, 31, 32,34	4/480	5/477	0.83 [0.24, 2.96]	0.778	0%	0.53
Cardiovascular History	Yes	29,31,34	76/1280	98/1230	0.80 [0.60, 1.07]	0.126	0%	0.69
	No	25,32	0/192	2/205	0.35 [0.04, 3.28]	0.354	0%	0.96
Baseline Creatinine	> = 1.5	25,29	17/333	28/339	0.63 [0.35, 1.12]	0.116	0%	0.68
	<1.5	24, 31,32,34	63/1572	76/1617	0.85 [0.62, 1.18]	0.338	0%	0.94
LDL Cholesterol Lowering	> = 30%	29	17/286	27/293	0.65 [0.36, 1.16]	0.142	-	-
	<30%	24,25,32	4/625	6/636	0.75 [0.23, 2.42]	0.625	0%	0.73
Follow-up time	> = 3	24, 29,31,32,34	80/1858	103/1910	0.80 [0.60, 1.06]	0.122	0%	0.90
	<3	25	0/47	1/46	0.33 [0.01, 7.81]	0.489	-	-

*Abbreviations*: *CI* confidential interval, *LDL* low density lipoprotein.

## Discussion

Statin is commonly used to lower LDL cholesterol levels in the general population, which reduces the risk of cardiovascular disease [8,13]. Moreover, many studies have shown the substantial benefits of statin therapy in patients with cardiovascular disease [36]. However, lack of information on the efficacy and safety of statin therapy in patients with CKD has limited the use of statins for these patients. We excluded patients with end-stage renal disease from our study, which is associated with extremely high cardiovascular morbidity and mortality rates, the co-morbidity of end-stage of renal disease, may be difficult to reverse in this phase [12,37]. This study was based on RCTs and investigated any possible correlation between statin therapy and the outcomes of any cardiovascular-related disease in patients with early to moderate renal disease (Kidney Disease Outcomes Quality Initiative [K/DOQI stage 1–4]).

This large quantitative review, including 42,426 individuals with a broad range of baseline characteristics, suggests that statin therapy could lead to a 24% reduction in the risk of cardiovascular disease, a 21% reduction in the risk of total mortality, and a 23% reduction in the risk of cardiovascular mortality in patients with mild to moderate CKD. A previous study indicated that statin therapy reduced the risk of cardiovascular disease, total mortality, and cardiovascular death in a subgroup analysis of pre-dialysis patients [15]. These findings were similar to the results of our study. Our main findings also support the conclusion of the Pravastatin Pooling Project (PPP 2004), which was a pooled analysis of three major statin studies on the subject level and demonstrated that statin therapy also appeared to reduce the incidence of cardiovascular disease and total mortality rate in patients with mild to moderate CKD [23]. Furthermore, our study also reported the results of myocardial infarction and stroke and concluded that statin therapy also contributed to a 34% reduction in the risk of myocardial infarction and a 30% reduction in the risk of stroke.

The outcome of cardiovascular disease, cardiovascular death, and total mortality was also reported in two previous meta-analyses [15,38]. Our meta-analysis extended this by including 8 additional randomized trials with patients across a wider background of cardiovascular risk [14,25,26,28-30,32,33]. We also performed more detailed subgroup analysis to explore whether the effects varied among different patients. We did not include trials with small sample size or short duration because cardiovascular events and CKD might not be clearly defined in these small trials, or the risk for outcome might not be accurate owing to insufficient observation times.

Some important factors such as baseline creatinine level, baseline GFR, and cardiovascular disease history may affect the results. The effects of statin therapy on cardiovascular disease in patients with baseline creatinine levels >1.5 mg/dL was not statistically significant. Similar effects were also found in a subgroup analysis of total mortality in patients with baseline creatinine levels >1.5 mg/dL, baseline GFR <60 mL · min^-1^ · 1.73 m^-2^, and a cardiovascular disease history. The results emphasized that statin therapy should be examined more carefully in these particular cases. However, further studies are needed to substantiate these conclusions.

A subgroup analysis of baseline LDL cholesterol levels and LDL lowering rates in patients with cardiovascular disease indicated no significant differences, although there were some benefits in the prevention of cardiovascular risk in total analysis. A possible reason could be that although LDL cholesterol level is considered an important risk factor for cardiovascular disease in patients, this strong correlation was attenuated by non-traditional cardiovascular risk factors such as the severity of kidney disease, oxidative stress, and inflammation in patients with renal insufficiency [32,33,36,39,40]. This may suggest that cardiovascular risk in patients with CKD, which is a complex mechanism, does not have a simple connection with dyslipidaemia, and may be a less effective indicator of dyslipidaemia as the major causal factor. Therefore, the benefits of statin therapy on cardiovascular disease in patients with mild to moderate CKD could not be explained by a differential action on lipid levels.

The effect of statin therapy on renal function could be qualitatively evaluated in nine included studies [24-26,28-33]. Five studies [14,24,31-33] have reported stable or no adverse effects on kidney function. Moreover, three trials [25,26,29] reported the renoprotective effect observed in the treatment group compared with the placebo group, and only one trial [27] reported adverse effects on kidney function. A possible reason could be that statin therapy reduces inflammation, which may slow the progression of tubulointerstitial nephritis, although a transient increase in proteinuria may be caused, and may protect against long-term renal damage [41]. Therefore, we may conclude that statin therapy has no serious adverse effects on kidney function, and is possibly beneficial to kidney function; however, these arguments definitely need further study with more detailed data on kidney function at the patient level.

Our analysis also found that statin therapy has no significant drug-related side effects such as rhabdomyolysis, cancer, or change in creatine phosphokinase levels compared with the control group. However, these conclusions might be unreliable because only a small number of trials were included in such subsets.

Our study also has some potential limitations. First, our result was based on published data, whereas individual patient data and original data were not available, which may limit the effective assessment of drug effects and safety. Second, the evaluation index of renal function differed in the included trials, which may affect the classification of CKD. Third, baseline characteristics such as the statin type, dosage, disease history, severity of CKD, and follow-up duration varied among included trials, which may have caused some heterogeneity. Last, publication bias was unavoidable in our study.

## Conclusion

In conclusion, our findings strongly suggest that statin therapy has significant benefits in preventing cardiovascular disease, stroke, myocardial infarction, and all-cause mortality in patients with mild to moderate CKD. Furthermore, our study also provides detailed evidence on the prevention of cardiovascular events in patients with different baseline characteristics and renal kidney function, which is needed for physicians to consider the results more carefully and comprehensively in clinical practice. We suggest that the ongoing trials be improved in the following ways: (i) promising interventions should be tested, including dosage, treatment duration, or a combination of these with influencing factors; (ii) details of adverse events of trials should be reported and recorded to allow evaluation of the side effects of any treatment in future trials.

## Competing interests

The authors declare that they have no competing interests.

## Authors’ contributions

Conception/Design: JH, XZ, Y-HZ. Collection and/or assembly of data: XZ, CX, Y-YQ. Data analysis and interpretation: XZ, Y-HZ, CX, AJ. Manuscript writing: XZ, Y-HZ. Final approval of the version to be published: JH, XZ, CX, Y-HZ, AJ, Y-YQ.

## Pre-publication history

The pre-publication history for this paper can be accessed here:

http://www.biomedcentral.com/1471-2261/14/19/prepub

## Supplementary Material

Additional file 1: Figure S1 Funnel plot of log relative risk vs. standard error of log relative risks. (a) Funnel plot for cardiovascular disease; (b) Funnel plot for total mortality; (c) Funnel plot for stoke; (d) Funnel plot for myocardial infarction; (e) Funnel plot for cardiovascular mortality. Click here for file
